# Gene set analysis methods applied to chicken microarray expression data

**DOI:** 10.1186/1753-6561-3-S4-S8

**Published:** 2009-07-16

**Authors:** Axel Skarman, Li Jiang, Hornshøj Henrik, Bart Buitenhuis, Jakob Hedegaard, Lene N Conley, Peter Sørensen

**Affiliations:** 1 Department of Genetics and Biotechnology, Faculty of Agricultural Sciences, Aarhus University, DK-8830 Tjele, Denmark

## Abstract

**Background:**

Gene set analysis is considered to be a way of improving our biological interpretation of the observed expression patterns. This paper describes different methods applied to analyse expression data from a chicken DNA microarray dataset.

**Results:**

Applying different gene set analyses to the chicken expression data led to different ranking of the Gene Ontology terms tested. A method for prediction of possible annotations was applied.

**Conclusion:**

Biological interpretation based on gene set analyses dependent on the statistical method used. Methods for predicting the possible annotations for genes with unknown function from the expression data at hand could be useful, but our results indicate that careful validation of the predictions is needed.

## Background

A major challenge in large-scale gene expression studies is the biological interpretation of the observed expression patterns. Gene set analysis identifies expression changes in functionally related genes and it is considered to be a way of improving understanding of the underlying biology [1]. Gene sets can be defined based on prior biological knowledge on gene functions available from public available databases (e.g. Gene Ontology (GO)) [2]. The aim of this work was to compare different gene set analyses methods when applied to a chicken microarray data set. As a high number of probes on the chicken microarray lack annotation we also applied a method to predict the possible annotations from the expression data.

## Methods

### The data – host reactions in broilers after a secondary challenge

The data originated from a microarray experiment conducted to study the host reactions in broilers shortly after a secondary challenge. The broilers were initially inoculated with phosphate buffered saline (P) or with *E. maxima *(M) followed by a secondary with PBS (P), *E. maxima *(M) or with *E. acervulina *(A), forming five challenge groups PP, PM, PA, MM and MA. Samples of the jejunum were collected 8 and 24 hours after the second challenge and gene expression profiles were obtained using chicken whole genome oligonucleotide microarrays. The result of the contrasts between MM8-PM8, MM8-MA8 and MM8-MM24 were provided for this workshop. A more detailed description of the experiment can be found in an adjacent paper [3]Hedegaard et al: "Methods for interpreting lists of affected genes obtained in a DNA microarray experiment".

### Gene Ontology class prediction

GO class predictions for genes with unknown GO annotations were based on expression ratios and support vector machine (SVM). SVM is a set of machine learning methods that can be used for data classification and has been implemented in Gist 2.3 version [4,5] that we have used in this study. The predictions were focused on significantly differentially expressed genes in the contrasts MM8-MA8, MM8-MM24 and MM8-PM8, defined as the probes with p-values at or below 0.05 after correcting for multiple testing by Benjamin and Hochberg's False Discovery Rate method (FDR) [6]. The total number of oligonucleotides representing differentially expressed genes was determined to be 2347. Gist requires expression ratio matrices without missing values, therefore the number of oligonucleotides were reduced to 936. Of these oligonucleotides, 280 oligonucleotides have previously been mapped to a GO Biological Process (BP) term. The expression ratios for these 280 oligonucleotides were defined as the training set. The test set for class prediction consisted of the expression ratios for the remaining 656 oligonucleotides without GO BP annotations.

### Defining gene sets for gene set analysis

Gene set analyses is based on the available annotation for the chicken genome. According to EADGENE Oligo Set Annotation Files [7] version 2 from 11^th ^of September 2008, there are 20460 unique oligonucleotides on the chicken array. Among these 14592 oligonucleotides represent 11532 Ensembl chicken genes. There are 2420 Ensembl chicken genes represented by multiple (2 to 9) oligonucleotides on the array.

Each of the gene lists for the three contrasts (MM8-MM24, MM8-MA8 and MM8-PM8) [4] contains 13158 oligonucleotides, of which 13126 are unique. The remaining 32 oligonucleotides are multiple copies of control probes. The oligonucleotides in the gene list were mapped to GO annotation with 3422 oligonucleotides associated with (BP), 4385 associated with molecular function (MF) and 3455 associated with cellular component (CC).

Gene sets were defined based on the annotated oligonucleotidesand gene sets with fewer than 5 oligonucleotides were excluded. There were originally 2553 BP, 1436 MF and 481 CC terms represented on the array. Applying the above criteria of gene set definition and filtering reduced this to 475 BP, 248 MF and 157 CC terms available for the analysis.

Since a unique gene can be represented by multiple different probes on a microarray, it is of interest to compare the gene set tests based on individual oligonucleotides (oligo-wise) or on individual genes (gene-wise).

### Gene set analysis methods and software

Gene set analysis was performed using software packages developed in Bioconductor [8] and R [9]. The tests used were the Wilcoxon test as implemented in the LIMMA package (version 2.14.5 [10,11]), Fisher's exact test [12] and Kolmogorov Smirnoff implemented in the topGO (version 1.8.1. [13]), and Globaltest [14,15] implemented in the Globaltest package (version 4.12.0). For the Fisher's exact test a predefined adjusted p-value of 0.05 was chosen to be the cutoff for individual oligonucleotides to be differentially expressed. Except for the Globaltest, the result of statistical hypothesis testing (t-statistics or p-value) for the oligonucleotides was used as input data for the gene set analyses. The Globaltest uses the expression data directly when testing gene sets and the corresponding p-values were computed using either the asymptotic distribution or permutations [14]. The gene set testing was done both with and without adjusting the p-values for multiple testing using FDR [6]. A two-sided binomial test was used to assess if the p-values from gene-wise and probe-wise gene set tests tended to be smaller in one case or another.

## Results and discussion

### Class prediction for genes with unknown GO annotations

We generated an expression ratio data set with 936 oligonucleotides for genes without missing expression values. These oligonucleotides were differentially expressed with false discovery rate at 0.05 in one or more of the contrasts (MM8-MM24, MM8-MA8 and MM8-PM8). From previous annotation, 280 oligonucleotides mapped to 467 GO BP terms and 656 oligonucleotides did not map to any GO BP term. We characterized two sets, one as a training set and one as a test set respectively. For each GO term in the training set it was predicted whether any of the oligonucleotides in the test set could be classified as belonging to the GO BP term based on similarity in expression profiles between the two sets across the tissue samples (MA8, MM8, MM24 and PM8).

In total, 301 out of 656 oligonucleotides were predicted to 104 GO BP terms with a discriminant value given by Gist above 1. Additional file 1 shows the top 20 predictions based on ranking the number of oligonucleotides for each GO BP term. For most GO BP terms the number of predicted oligonucleotides is higher than the number of oligonucleotides that was previously mapped. Additional file 2 shows an example of class prediction for the GO term 'immune response' (GO:0006955) visualized by hierarchical clustering of the expression ratios for genes previously known to map to this GO BP term and genes that were predicted to belong to this GO term. Validation of the prediction method using this GO BP term was attempted using the following approach. Each oligonucleotides with previously known mapping to this term was taken out one by one and tested whether class prediction classified it to the expected term. Unfortunately, validation was only possible for oligonucleotides ID RIGG20020 with the combination of tissue expression data and prediction method used in this study. The class prediction approach used in this study was inspired by a previous study in mice [5]. In comparison to our study, they used a much higher number of different annotated GO terms and a much higher number of different tissues, which may explain the low validation success that we observed here.

### Adjustment for multiple testing

To adjust for multiple testing we applied FDR to the different gene set tests. After adjustment most methods had no terms with p-value below 0.05 for all categories of GO terms, the exception being Globaltest. Except for Global test the corrected p-values were in most cases close to one or relatively high. There were even many cases where all p-values became one or close to one. It was therefore decided to compare the different gene set methods without adjusting for multiple testing. In our attempt to account for multiple testing we ignored the structure of the GO graph. Multiple testing procedures assume that the tests performed are independent. This assumption is often violated as the GO terms are not independent to each other due to hierarchical structure of GO and the usage of multiple GO terms in the annotation of one gene. Another problem with most multiple testing adjustments is that they do not change the ranks and therefore the relative importance of the different GO terms. Alternatively it is possible to use an adjustment method that account for the dependencies. One example could be to use the focus level procedure [16] even though it is not valid for all kinds of tests.

### Gene set tests based on individual oligonucleotides or genes

Since the same gene can be measured by a set of different oligonucleotides which are very likely to have the identical annotation, it is of interest to compare the gene set tests based on individual oligonucleotides (oligo-wise) or on individual genes (gene-wise). For this comparison we used the Wilcoxon test which determines if the oligonucleotides/genes in the set is up-regulated (up), down-regulated (down) or differentially expressed regardless of direction (mixed). Gene sets were defined based on GO annotation and we analyzed all three contrasts (MM8-MM24, MM8-MA8 and MM8-PM8).

**Figure 1:**
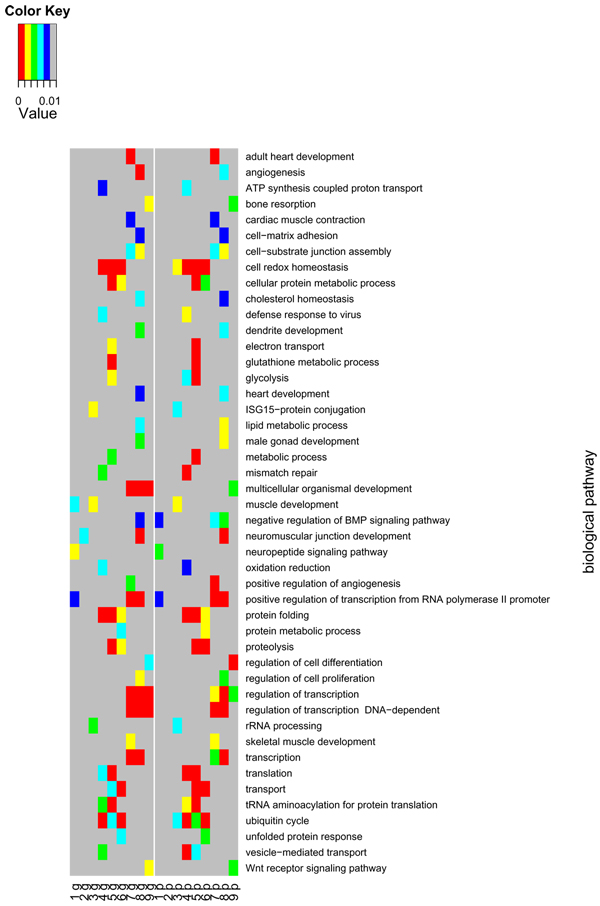
**Visualization of class predictions for GO BP term 'immune response' showing expression ratio profiles for both oligonucleotides with known mapping to this term (1) and oligonucleotides predicted to belong to this term (0)**. Validation was possible only for oligonucleotides ID RIGG20020.

We computed the Spearman correlations between the vectors of p-values from Wilcoxon tests based on individual oligonucleotides or genes. For the Wilcoxon test the direction was not considered. Each vector was generated in the gene set test defined by GO class for each contrast. There was a high correlation (0.84–0.92) between these tests. Comparing across all gene set tests it appeared that the gene-wise p-values in general are smaller than the oligo-wise p-values (smaller in 58% of the cases corresponding to p = 2.2*10^-16 ^in a binomial two sided test). Figure 1 shows the BP terms tested (up-regulated, down-regulated or differentially expressed) having a p-value less than 0.01 for the different contrasts. The pattern of BP terms for gene-wise and oligo-wise tests is very similar across the different contrasts although there are cases where terms for only the gene-wise or the oligo-wise test.

For the tests based on individual genes the median value of the t-statistics from all its corresponding oligonucleotides was used. The median value measures the trend of all its transcripts as a whole and if the different oligonucleotides measure different splice variants using the median may lead to biased results. Alternatively, the t-statistic for a single oligonucleotides could be used to represent the gene in the gene set test. Although the results indicate only minor differences between gene set test based on individual oligonucleotides or genes it is difficult to generalize to other datasets. The best choice depends on the design of the array and in particular if the number of replicate probes varies for different genes it will often be better to use tests based on individual genes.

### Gene set tests taking GO structure into account

**Figure 2:**
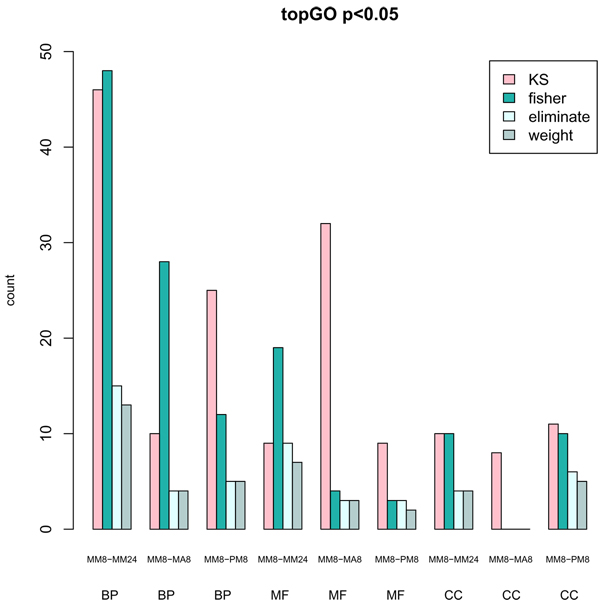
**All the BP gene sets which were tested (up-regulated, down-regulated or differentially expressed) having a p-value less than 0.01 are shown for the different contrasts**. g: stands for gene-wise and p: stands for oligo-wise. 1: differentially expressed BP gene sets from MM8_MM24; 2: differential expressed BP gene sets from MM8_MA8; 3: differentially expressed BP gene sets from MM8_PM8; 4: up regulated BP gene sets from MM8_MM24; 5: up regulated BP gene sets from MM8_MA8; 6: up regulated BP gene sets from MM8_PM8; 7: down regulated BP gene sets from MM8_MM24; 8: down regulated BP gene sets from MM8_MA8 and 9: down regulated BP gene sets from MM8_PM8.

GO has a hierarchical structure that forms a directed acyclic graph which leads to a high degree of dependencies between the tested terms. We used the topGO package which based on the Fisher's exact test implements two algorithms (eliminate and weight) that takes the GO structure into account when testing the gene sets. These two algorithms were compared to the "classical" Fisher's exact test and the Kolmogorov Smirnoff test which both ignores the GO structure. The terms for the three GO categories were tested for the three contrasts. Figure 2 shows that taking the GO structure into account leads to fewer terms with p-value below 0.05 which may indicate increased specificity as suggested by the authors of the method [13].

### Comparing Globaltest and Wilcoxon test

We also compared the results from the Globaltest and Wilcoxon test. These methods differ in two important aspects. First, Wilcoxon test is a competitive test in the sense that genes in the set are compared to other genes whereas Globaltest is a self-contained test which generally leads to higher power [17]. Second Wilcoxon test treats the individual genes/oligonucleotides as the sampling units and Globaltest treats the subjects as sampling units which is a more sound statistical approach. These differences may in part explain when comparing different methods the highest ranked terms are not necessarily the same. We compared the two methods by the number of common terms among the terms with p-value below 0.05 or among the 15 top ranking terms (Table 1).

**Table 1:** Comparison of Wilcoxon and Globaltest.

	Wilcoxon (p-value < 0.05)	Global Asymptotic			Global Permutation	
		
		p-value < 0.05	Common with wilcoxon in top 15	Common with wilcoxon (p-value < 0.05)	p-value < 0.05	Common with wilcoxon (p-value < 0.05)
MM8_MM24	17	228	3	16	285	17
MM8_MA8	22	215	3	21	264	20
MM8_PM8	30	383	4	30	400	30

For Wilcoxon and Globaltest with the two p-value estimation methods the numbers of terms with p-value below 0.05 are given. The number of terms with p-values below 0.05 for each method and overlapping between Wilcoxon and Globaltest are shown as well. For Globaltest with Asymptotic p-value calculation the top 15 terms overlapping with the top 15 for Wilcoxon are shown.

The Globaltest gave a much larger number of terms with a p-value below 0.05. It is however unclear to what extent this comes at a cost of a higher number of false positives. For each of the comparisons the terms with p-value below 0.05 given by the Wilcoxon test were almost all found by the Globaltest (p-value below 0.05). However, only 3 or 4 out of the top 15 terms are overlapping. For the Globaltest we also found that the number of terms with p-values below 0.05 to be higher when p-values were computed using permutation compared to when the asymptotic was used.

## Conclusion

Applying different gene set test to the EADGENE chicken expression data led to different ranking of the GO terms tested. Therefore biological interpretation based on gene set analyses is depending on the statistical method used. Methods for predicting the possible annotations for genes with unknown function from the expression data at hand are useful, but our results indicate that careful validation of the predictions is needed.

## List of abbreviations used

GO: Gene Ontology; SVM: Support Vector Machine; FDR: Benjamin and Hochberg's False Discovery Rate; BP: Biological Process; MF: Molecular Function; CC: Cellular Component;

## Competing interests

The authors declare that they have no competing interests.

## Authors' contributions

The authors contributed equally to the final manuscript.

## Supplementary Material

Additional file 1 **Top 20 class predictions for GO BP ranked descending by the number of predicted oligonucleotides**. The table contains the following columns: GO ID: GeneOntology Identifier, GO description: GeneOntology description, Oligonucleotide count known: number of oligonucleotides that were previously mapped to the GO BP term, Oligonucleotide count predicted: number of oligonucleotides that were predicted to belong to the GO BP term. Click here for file

Additional file 2 **GO BP term ‘immune response’ showing expression ratio profiles for both oligonucleotides with known mapping to this term (1) and oligonucleotides predicted to belong to this term (0).** Validation was possible only for oligonucleotides ID RIGG20020. Click here for file
