# Supplementary material for: Gene set analysis methods applied to chicken microarray expression data
Source: BMC Proc. 2009 Jul 16;3(Suppl 4):S8. doi: 10.1186/1753-6561-3-S4-S8 (PMC2712751; doi:10.1186/1753-6561-3-S4-S8)
Supplement: Additional file 1 — Top 20 class predictions for GO BP ranked descending by the number of predicted oligonucleotides. The table contains the following columns: GO ID: GeneOntology Identifier, GO description: GeneOntology description, Oligonucleotide count known: number of oligonucleotides that were previously mapped to the GO BP term, Oligonucleotide count predicted: number of oligonucleotides that were predicted to belong to the GO BP term. [file 1753-6561-3-S4-S8-S1.pdf]

Top 20 class predictions for GO BP ranked descending by the number of predicted oligonucleotides

GO ID	GO description	oligonucleotide count known	Oligonucleotide count predicted
GO:0030154	cell differentiation	12	23
GO:0009966	regulation of signal transduction	3	22
GO:0007169	transmembrane receptor protein tyrosine kinase signaling pathway	3	21
GO:0007155	cell adhesion	9	19
GO:0007049	cell cycle	8	18
GO:0006955	immune response	7	17
GO:0007010	cytoskeleton organization and biogenesis	2	17
GO:0045892	negative regulation of transcription DNA-dependent	5	17
GO:0006412	Translation	11	16
GO:0006468	protein amino acid phosphorylation	13	16
GO:0001578	microtubule bundle formation	2	14
GO:0006281	DNA repair	4	14
GO:0008285	negative regulation of cell proliferation	8	14
GO:0016055	Wnt receptor signaling pathway	5	14
GO:0007417	central nervous system development	3	13
GO:0051017	actin filament bundle formation	2	13
GO:0006094	Gluconeogenesis	4	12
GO:0007165	signal transduction	22	12
GO:0007275	multicellular organismal development	17	12
GO:0008284	positive regulation of cell proliferation	8	12

Oligonucleotide count known is the number of oligonucleotides that were previously mapped to the GO BP term and oligonucleotide count predicted is the number of oligonucleotides that were predicted to belong to the GO BP term.
