# Supplementary material for: Gene set analysis methods applied to chicken microarray expression data
Source: BMC Proc. 2009 Jul 16;3(Suppl 4):S8. doi: 10.1186/1753-6561-3-S4-S8 (PMC2712751; doi:10.1186/1753-6561-3-S4-S8)
Supplement: Additional file 2 — GO BP term ‘immune response’ showing expression ratio profiles for both oligonucleotides with known mapping to this term (1) and oligonucleotides predicted to belong to this term (0). Validation was possible only for oligonucleotides ID RIGG20020. [file 1753-6561-3-S4-S8-S2.pdf]

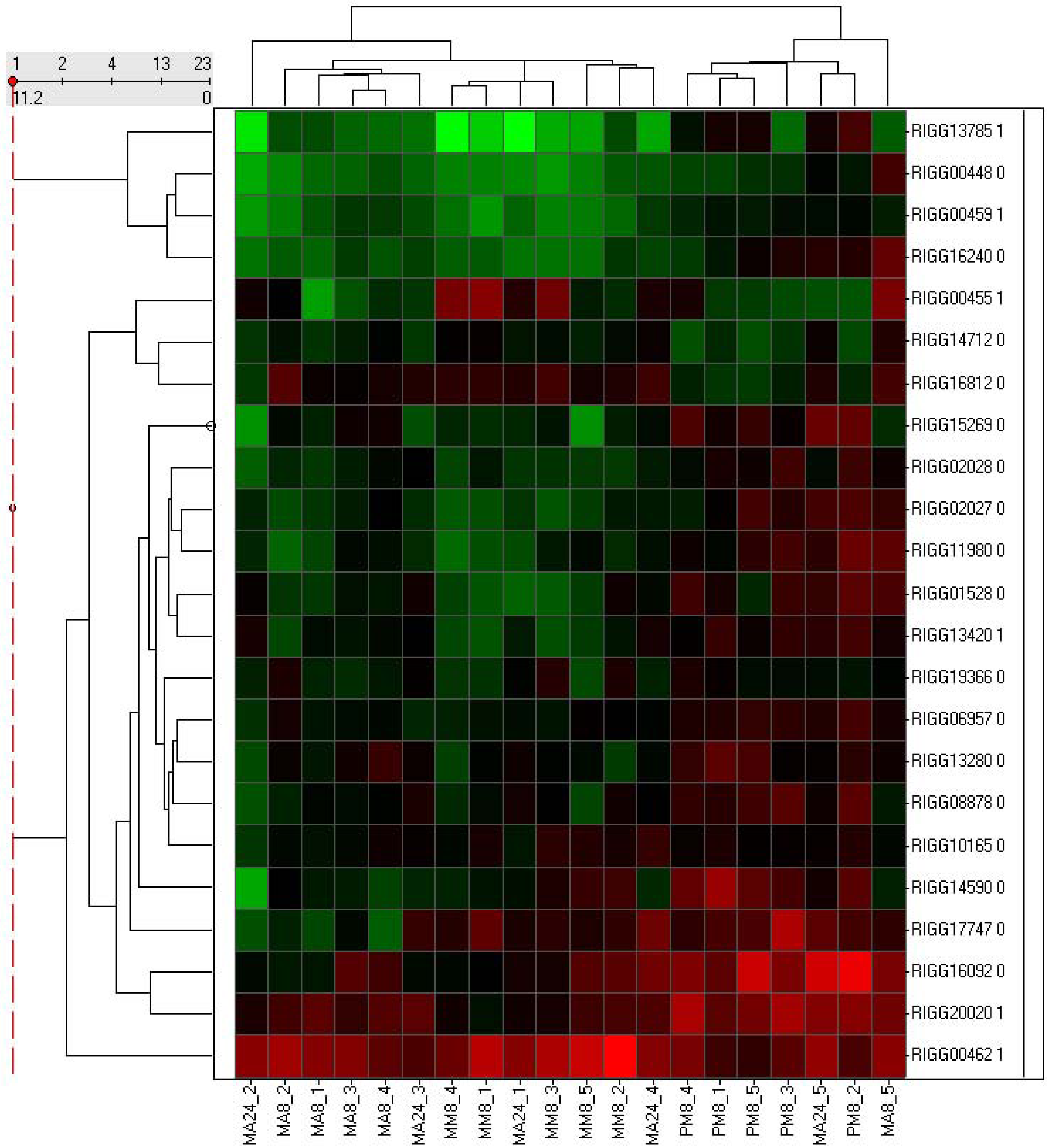

Visualization of class predictions. GO BP term 'immune response' showing expression ratio profiles for both oligonucleotides with known mapping to this term (1) and oligonucleotides predicted to belong to this term (0). Validation was possible only for oligonucleotides ID RIGG20020.
